# Crystal Growth of Cubic and Hexagonal GaN Bulk Alloys and Their Thermal-Vacuum-Evaporated Nano-Thin Films

**DOI:** 10.3390/mi12101240

**Published:** 2021-10-13

**Authors:** Marwa Fathy, Sara Gad, Badawi Anis, Abd El-Hady B. Kashyout

**Affiliations:** 1 Electronic Materials Department, Advanced Technology and New Materials Research Institute, City of Scientific Research and Technological Applications (SRTA-City), Alexandria 21934, Egypt; akashyout@srtacity.sci.eg; 2 Spectroscopy Department, Physics Division, National Research Centre, 33 El Bohouth St., Dokki, Giza 12622, Egypt; badawi.ali@gmail.com; 3 Molecular and Fluorescence Lab., Central Laboratories Network, National Research Centre, 33 El Bohouth Str., Dokki, Giza 12622, Egypt

**Keywords:** GaN, crystal growth, cubic and hexagonal structure, blue and yellow luminescence, electron lifetime

## Abstract

In this study, we investigate a novel simple methodology to synthesize gallium nitride nanoparticles (GaN) that could be used as an active layer in light-emitting diode (LED) devices by combining the crystal growth technique with thermal vacuum evaporation. The characterizations of structural and optical properties are carried out with different techniques to investigate the main featured properties of GaN bulk alloys and their thin films. Field emission scanning electron microscopy (FESEM) delivered images in bulk structures that show micro rods with an average diameter of 0.98 µm, while their thin films show regular microspheres with diameter ranging from 0.13 µm to 0.22 µm. X-ray diffraction (XRD) of the bulk crystals reveals a combination of 20% hexagonal and 80% cubic structure, and in thin films, it shows the orientation of the hexagonal phase. For HRTEM, these microspheres are composed of nanoparticles of GaN with diameter of 8–10 nm. For the optical behavior, a band gap of about from 2.33 to 3.1 eV is observed in both cases as alloy and thin film, respectively. This article highlights the fabrication of the major cubic structure of GaN bulk alloy with its thin films of high electron lifetime.

## 1. Introduction

Generally, gallium nitride (GaN) is one of the group III-V nitrides, with a band gap ranging between 3.27 eV and 3.47 eV [1]. At room temperature, it has two different phases of structural properties and dislocations: wurtzite (2H) and zinc-blende (3C). The cubic phase (c-GaN) is metastable, making its growth a difficult challenge. It is *p*-type doping and has higher mobility than hexagonal structure. Cubic or zinc-blende structure is easily cleavable, which, combined with the evidence for optical gain, offers attractive possibilities such as blue light emission and promises to achieve improved efficiencies for green-wavelength LEDs [2]. Hexagonal phase (h-GaN) is a promising candidate for creating high power devices due to its large band gap (3.47 eV) with high saturation velocity. Furthermore, spontaneous piezoelectric polarization occurs in h-GaN-induced internal electric fields, which causes energy band tilting [3] and influence optoelectronic devices’ performance [4].

Many studies fabricated GaN using different physical growth techniques such as metal organic chemical vapor deposition (MOCVD) [5], ion-beam-assisted molecular beam epitaxy [6], reactive molecular beam epitaxy [7], thermal ammonization [8], physical vacuum vapor deposition [9], chemical vapor deposition (CVD) [10,11], thermal vapor deposition [12], and combustion method [13,14,15].

MOCVD is the most useful technique in the preparation of high-quality h-GaN wurtzite phase, although it is not suitable for c-GaN because the process is done at high temperature. By using lower-temperature techniques such as molecular beam epitaxy, the percentage of h-GaN in the thermodynamically stable phase could be dominant. Furthermore, the structural and optical properties of the deposited GaN depend on the type of substrate. [1]

This work involves the synthesis of high-quality GaN bulk alloy as a mixture of cubic and hexagonal microstructure using a simple technique, “crystal growth”, under a temperature of 850 ^°^C from Ga metal with pure ammonia gas, and demonstrates the structure transformation process due to the effect of the deposition process using high thermal vacuum evaporation on a glass substrate.

## 2. Materials and Methods

**Figure 1:**
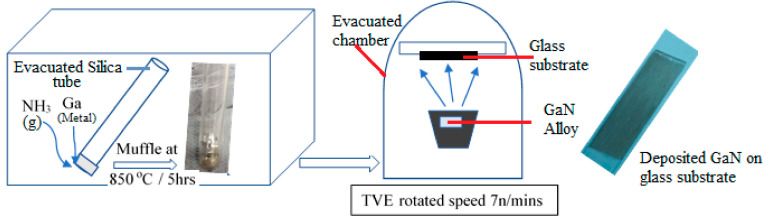
Scheme diagram of the experimental work.

The following chart explains the experimental procedure carried out through this work (Figure 1).

For GaN bulk alloy preparation, gallium metal of 99.999% purity (Aldrich-Sigma, Saint Louis, MO, USA) is introduced in a silica tube, which is first vacuumed at 10^−4^ Torr and then filled with pure NH_3_ gas (99.9%). The tube is finally sealed by welding a narrow nick, which is made to insert the NH_3_ gas. The ammonia flow rate is kept at 0.1 sccm. The tube temperature reaches 850 °C for 2 h in a muffle furnace (Carbolite AAF117) and then is reduced inside the furnace in order to prevent the segregations previously detected in InGaN alloys [16,17].

For GaN thin film using thermal vacuum evaporator, the vapor faces the glass substrates and condenses as a thin film. The glass substrate is fixed at the substrate holder, which is rotated at a fixed speed during the evaporation process. Finally, the GaN is deposited on the glass substrate with a thickness of 119 nm (measured from SEM cross section). The structural and morphological properties of the GaN thin film are systematically analyzed by X-ray diffraction (XRD-Shimadzu XRD 7000 maxima powder diffractometer, Kyoto, Japan), field emission scanning electron microscopy (FESEM-Quanta 250, USGS, Laurel, MD, USA), photoluminescence (PL-Perkin Elmer Luminescence Spectrometer Model LSS, Shared Instrumentations, Richmond, CA, USA), transmission electron microscopy (JEM-2100, JEOL, Japan) running at 200 KV. Also, we report triggered single-photon emission from gallium nitride with nanostructure by using fluorescence lifetime imaging microscopy (FLIM system Alba with v5 from ISS). A laser diode of 640 nm was used for the excitation, coupled with a scanning module of (ISS) through multi-band dichroic filter to epifluorescence microscope (Model IX73, Olympus, Tokyo, Japan) with UPLSA 60X objective 1.2 NA and 0.28 mm width. Emission is observed and detected by cooled low noise (below 100 counts/s) with a detector of GaAs fast PMTs for time-correlated single-photon counting (TCSPC). FLIM data are acquired using ISS A330 Fast FLIM module with n harmonics of 20 MHz laser repetition frequency. FLIM data are analyzed with Vista Vision Suite software (Vista v.204 from ISS). The FLIM analysis is carried out using the fitting indicating the formation of cubic structure. The XRD peaks at 60.8° (301), 57.33° (110), 37.9° (101) and 33.39° (100) are for JCPDS Card No. [01-079-2499], which presents the hexagonal structure. In general, the common structure algorithm and the phasor analysis are used with the hexagonal phase.

## 3. Results and Discussion

### 3.1. XRD

**Figure 2:**
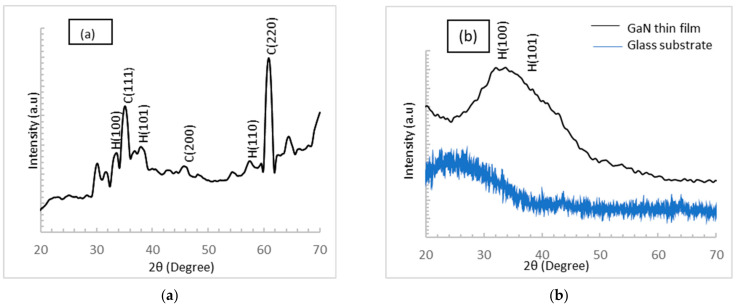
The XRD spectra of GaN (**a**) bulk alloy and (**b**) thin film on glass substrate.

Figure 2 shows the XRD spectra of the bulk alloy and thin film of GaN. The direct reaction of Ga-melt with NH_3_-gas at a temperature of 850 °C gives rise to the formation of micro-crystalline GaN. As shown in Figure 2a, the XRD peaks at 37.9° (101) and 33.39° (100) are for JCPDS Card No. [01-076-0703] [1], and the cubic phase is prepared with special conditions.

For bulk alloy, the structural quality of cubic GaN with low hexagonal phase was determined, and the percentage of cubic to hexagonal structure is 80% according to Equation (1) below [18]:

*X_C_* =1−[1/(1+1.26(*I*_c_/*I*_h_))]^−1^ (1)

where *Xc* is the weight fraction of cubic structure in the mixture, and *I*_c_ (220) and *I*_h_ (110) are the diffraction peak intensities of the cubic and hexagonal structure, respectively.

There is a shift in XRD theoretical value peaks from the experimental peaks due to the strain from scattered intensity distribution [2]. According to Reference [2], the lattice parameters of GaN are; *a* = 0.3232 nm and *c* = 0.5269 nm, so the unit cell *c*/*a* = 1.630, but in this work, *c*/*a* = 1.3; lattice constants *a* and *c* can be obtained from the Bragg diffraction formula (Equations (2)–(4)) [2]:

2d sin θ = nλ(2)

(3) a=dhkl[(3/4h^2+hk+k^2+l^2 a/c2]0.5

(4) c=dhkl[(3/4h^2+hk+k^2+l^2 c/a2]0.5

where n as an integer is the “order” of the reflection, λ is the incident X-rays of the wavelength in nm, d is the inter-planar spacing of the crystal in Å, θ is the incidence angle in radians, *d*(*hkl*) is the inter-planar distance, and *h*, *k*, *l* are the values of Miller Indices.

Figure 2b shows the diffraction pattern of thin film that is deposited on glass substrate (XRD of glass substrate shown in Figure 2b as blue line) using thermal vacuum evaporation. A broad diffraction peak with semi-crystalline structure appears at 2θ of 31° to 33.88° (100) and another peak at 38.40° (101); JCPDS Card No. [01-079-2499] refers to GaN with hexagonal structure with no appearance of cubic structure. This result is dependent on the vapor–solid (VS) mechanism in the vacuumed chamber of the thermal vacuum evaporator [19]. The high temperature helps in the transformation of cubic structure into h-GaN layers [19].

### 3.2. EDX Analysis

Table 1 shows the EDX analysis for the produced GaN bulk alloy prepared by crystal growth technique at 850 °C and GaN thin film. For the GaN bulk alloy, EDX data confirm the existence of Ga and N elements, indicating the formation of GaN structure (the reaction mechanism is shown in Equations (5) and (6)) and form an alloy with Ga:N composition of 1:1.

2NH_3_(g) →N_2_(g) + 3H_2_(g) (5)

2Ga(s) + N_2_(g) → 2GaN(s) (6)

After the deposition of GaN alloy on glass substrate using thermal evaporation technique, it can be seen that the atomic percentage of nitrogen increases due to thermal decomposition of GaN at high deposition temperature [20].

### 3.3. FESEM of GaN

#### GaN Bulk Alloy

**Figure 3:**
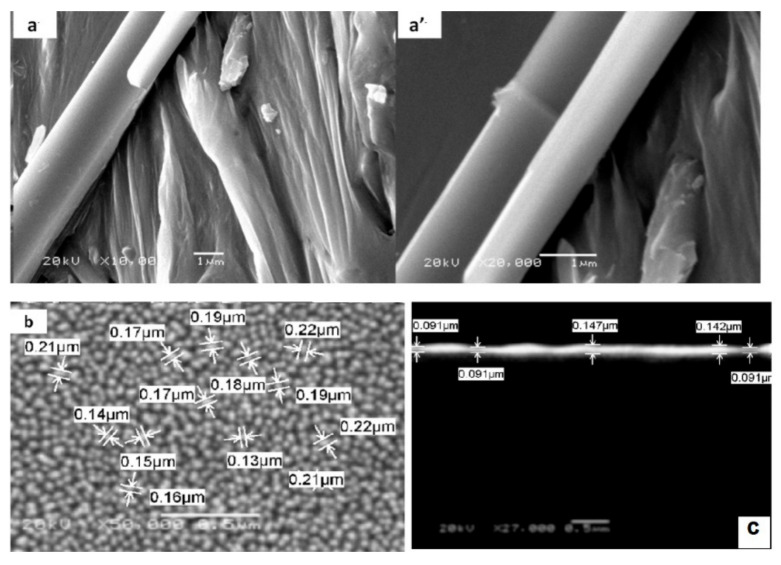
FESEM of GaN; (**a,a’**) bulk alloy, (**b**) thin film, and (**c**) cross-section area of the thin film.

The morphology of the GaN bulk alloy and its thermal-vacuum-evaporated thin film is investigated using field emission scanning electron microscopy (as shown in Figure 3). For GaN bulk alloy (Figure 3a), uniform micro-rods with an average diame-ter of 0.98 µm are observed. In some places, these microrods clearly coalesce to form few micron-size GaN bundles with lengths up to 30 µm. It shows that the growth of GaN alloy on silica tube yields rough and regular surface morphologies.

After the thermal evaporation deposition process, microrods convert to uniform microsphere particles with diameter ranging from 0.13 µm to 0.22 µm (as shown in Figure 3b). The mechanism of the thin film deposition may occur in the following stag-es: nucleation stage, fast attachment to form micro-rods network, growth of mi-cro-wires accompanied by fragmentation of network, and cleaving of spherical-like particles [21]. The cross-sectional view shown in Figure 3c of the thin film shows two parallel surface structures. Furthermore, there are no peels and cracks on the surface. The average thickness of the thin film as shown in Figure 3c is about 100 nm. A well-adhered and high film coverage on the glass substrate is clearly evident, as shown in Figure 1 for the GaN thin film.

### 3.4. Transmission Electron Microscopy

**Figure 4:**
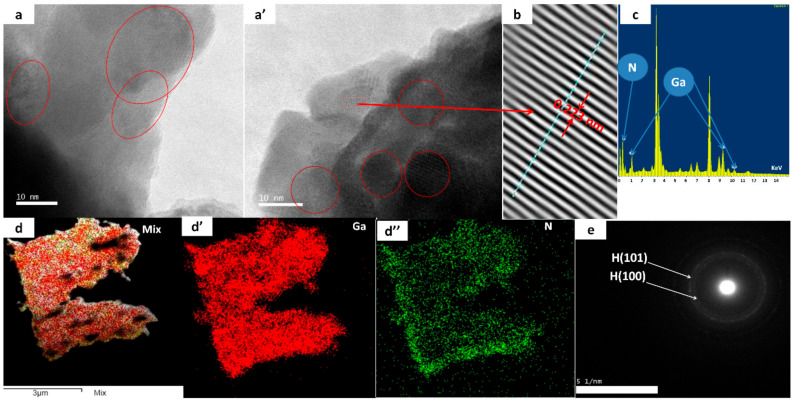
TEM and HRTEM of GaN; (**a**,**a’**) thin film, (**b**,**b’**) d-spacing, (**c**) EDAX spectrum, (**d**,**d’**,**d’’**) Ga and N mapping, and (**e**) SAED patterns.

The microstructural properties of GaN thin film deposited on glass substrate are further investigated by TEM (as shown in Figure 4). We detached the GaN film to prepare a TEM specimen by scratching a thin layer and dispersing it in ethanol. The surface morphology of GaN thin film shows nanoparticles with spherical nature with size around 8-10 nm. The nano-crystallization process may be explained by the “propagation” of the SFs network. It probably occurred because of the highly strained area indicated in the XRD data of the thin film. This strained area is below the SFs that are sensitive to the lattice disorder, as they appeared in HRTEM. This growth of GaN nanoparticles resembles overgrowth by MOCVD and PLD [22,23,24]. The lattice spacing is 0.223, which is a reflection of the (100) plane of h-GaN, as shown in Figure 4b. Ga and N elements are distributed regularly across the particles, as shown in Figure 4d,d’,d”. Selected-area electron diffraction (SAED) shows (Figure 4e) semi-crystalline behavior, as indicated by the regular rings.

### 3.5. Optical Properties of GaN as a Bulk and as Thin Film

Photoluminescence (PL) measurements are commonly applied techniques for the qualitative investigation of both GaN bulk alloys and thin films to detect material defects [25]. These native defects, which are present in semiconductor materials, arise from either non-stoichiometric crystal growth or an annealing process and consequently affect the electrical and optical properties of these materials [22]. The dissociation of nitrogen element during the reaction procedures as shown in Equations (5) and (6) may lead to the generation of the crystallographic defects and results in either nitrogen vacancy VN or gallium vacancy VGa, rather than a change in impurities [26]. GaN has two peaks of luminescence emissions; the yellow luminescence (YL) band centered at 2.1 eV (590 nm)–2.3 eV (539.13 nm) and the blue luminescence (BL) band centered at about 3.1 eV (401.3 nm) are generally considered related to defects in GaN [25].

**Figure 5:**
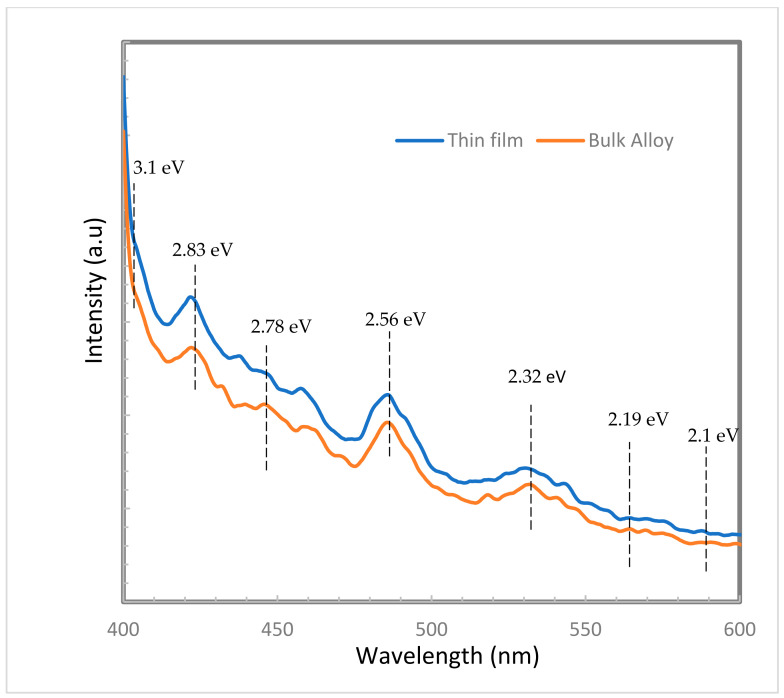
Bandgap of GaN bulk alloy and thin film.

The rate of dissociation is low enough to be compensated for by the NH_3_ ambient gas, yielding a small amount of native defects in the GaN microcrystals, which is responsible for the difference in emission peaks. PL measurements for the GaN bulk alloy and thin film deposited on glass substrate are presented in Figure 5. As shown in this figure, the PL graph of the GaN bulk alloy has near-wavelength emission (NBE); blue (BL) and yellow emission (YL) at wavelengths of 404.4 nm (3.1 eV) to 533.5 nm (2.32 eV), respectively. The luminescence of (BL) and (YL) may be due to the presence of Ga vacancies, dislocations [17], and amorphous phases [25]. The intensity of the 3.1 eV band is related to a significant presence of stacking faults or dislocations and point defects in the samples [27].

The peak at 438.5 nm (2.83 eV) may originate from the luminescence coming from the dissociation of excitons bound to neutral donors, according to Reshchikov and Morkoc [22]. The PL spectrum of the bulk alloy also reveals the presence of a BL at both 457.48 nm (2.71 eV) and 438.5 nm, which are be attributed to gallium and nitrogen vacancies and deep-level impurities [25].

For GaN thin film, the sample shows many emission peaks ranging from 533.5 nm to 404.4 nm with a slightly blue shift. The reason for the blue shift is the ionization of the bound excitons as the deposition temperature increases, resulting in recombination of free excitons, causing the resultant blue shift [28]. A luminescence centered at around 446.48 nm (2.78 eV) is associated with a broad shoulder at 457.48 nm (2.71 eV). The peak at 485.07 nm (2.56 eV) is a broad and intense green band that is associated with the luminescent center produced at the dislocation edges originating from both Ga and N vacancies. It also could be explained by the gallium vacancy, as seen in the bulk that appears in hexagonal and cubic phases.

In general, the intensity of BL decreases quickly, while the intensity of YL shows a slight decrease, and its peak energy declines greatly. BL intensity slightly decreases, and its energy peak falls slightly with temperature increase through the deposition process. However, YL intensity is increased when the peak energy is reduced (as shown in Figure 5).

The optical transient behaviors of BL or YL in GaN have been reported in the literature before [17,29]. There may be two main explanations for their optical transient phenomena. First, the Coulomb fields, which are caused by the charge-trapping centers, may block the diffusion of carries to BL-related (or YL-related) defects [30,31]. This enhancement in the shield effect will cause decreases in BL and YL as a result of the evaporated high temperature. A second explanation may arise from the transformation of the meta-stable point defects through the recombination process, enhancing the defect reaction mechanism, which appeared according to the cubic phase’s meta-stable property, as shown in the XRD data [25].

### 3.6. Time-Resolved Analysis

**Figure 6:**
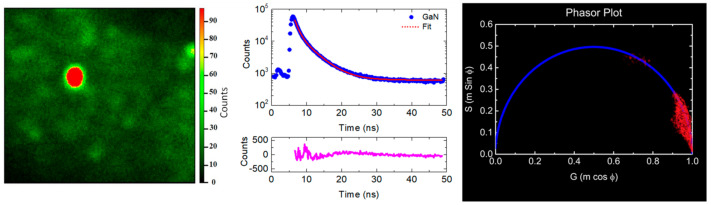
The raw FLIM data for GaN. The middle panel shows the photoluminescence intensity decay with the fitting curve. The curve at the bottom panels is the fitting residual. The left panel in is the phasor plot representations from fluorescence FLIM data.

Figure 6 shows the raw FLIM data for GaN, with photoluminescence intensity decay curve of the nano-crystals. The decay curve is fitted by the sum of two exponential functions, with more than 97% of counts having 1.12 ns lifetime and the rest having 4.2 ns. The right panel of Figure 6 shows the phasor plot for the GaN. The intensity of the phasor plot decays for the corresponding FLIM image, as shown in the left panel of Figure 6. In this plot, every pixel in the image is normally represented in a 2D diagram with two coordinates; namely, S and G. These two coordinates are based on the phase shift (φ) between the transmitted wave and the resulting PL wave and demodulation factor (m) in the lase source. The S and G components are given by the following Equations (7) and (8) [32]:

(7) $$S=\mathrm{msin}\left(\varphi \right)=\frac{\omega \tau }{1+\omega^{2\;}\tau^{2}}$$

(8) $$G=m\cos\left(\varphi \right)=\frac{1}{1+\omega^{2\;}\tau^{2}}$$

where *τ* is the electron lifetime and *ω* = 2*πf* is the laser modulation angular frequency (20 MHz). From Equations (7) and (8), the lifetime $\tau =\left(\frac{1}{\omega }\right)\frac{S}{G}$. The phasor plot for the GaN shows a cluster of points located at the edge of the phasor plot semicircle, indicating that the GaN decay is single-exponential decay with an average life-time of 1.12 ns as calculated from Equations (7) and (8).

## 4. Conclusions

In this study, a new line in GaN emission shifted to red light. The preparation of the bulk and thin film is very simple and economic. The bulk alloy preparation is a new technique for the generation of a III-nitride group depending on the crystal growth temperature. Furthermore, it was simple to use thermal vacuum evaporation to achieve this the target, and it is also dependent on the passing current and the deposition time. Furthermore, it plays an important role of eliminating the oxidation to obtain a pure GaN thin film. The crystal growth occurs in two phases apparent in XRD diffraction peaks and from PL. This thin film could be applied as a window material or emitter in some solar devices. From XRD and TEM investigation, we detected the formation of a strained region beyond the SFs network, which may be due to the point defect clustering and a band of planar defects that appeared in the surface. Time-resolved analysis reveals that PL intensity decays mainly in 1.12 ns, while the rest of the counts decay in 4.2 ns. Finally, these results support the use of a thermal evaporator for the growth of nano GaN particles used in short light-emitting diodes.

## Figures and Tables

**Table 1:** The EDX analysis of GaN bulk alloy and thin film.

Sample	Ga%	N%
Bulk alloy	50.8	49.2
Thin film	44.13	55.87
